# Longitudinal Monitoring of Metabolic Gradients in Microreactor Culture Platforms by Raman Spectroscopy

**DOI:** 10.3390/bios16050266

**Published:** 2026-05-02

**Authors:** Maitane Márquez, Javier Plou, Stefan Merkens, Eneko Lopez, Carla Solé, Esther Arnaiz, Mariana Medina-Sánchez, Charles H. Lawrie, Andreas Seifert

**Affiliations:** 1 Nanoengineering Group, CIC nanoGUNE BRTA, Tolosa Hiribidea 76, 20018 San Sebastian, Spain; 2 Molecular Oncology Group, Biogipuzkoa Health Research Institute, Paseo Dr. Begiristain s/n, 20014 San Sebastian, Spain; 3 Nanobiosystems Group, CIC nanoGUNE BRTA, Tolosa Hiribidea 76, 20018 San Sebastian, Spain; 4 Center for Molecular Bioengineering (B CUBE), Dresden University of Technology, 01307 Dresden, Germany; 5 IKERBASQUE, Basque Foundation for Science, Euskadi Plaza 5, 48009 Bilbao, Spain; 6 Radcliffe Department of Medicine, University of Oxford, Oxford OX3 9DU, UK; 7 Sino-Swiss Institute of Advanced Technology (SSIAT), Shanghai University, Shanghai 201899, China

**Keywords:** metabolic heterogeneity, Raman spectroscopy, microreactors, real-time monitoring

## Abstract

Metabolic heterogeneity within the cell microenvironment is a key driver of cancer progression and resistance to therapy. However, current approaches lack the spatial and temporal resolution required to capture its dynamics in living systems. While recent advances in 3D cell culture models and metabolomic profiling have improved our understanding of the tumor niche, their integration with real-time optical sensing remains underdeveloped. Here, we present an integrated platform combining a 3D-printed microreactor culture chamber with Raman spectroscopy to enable non-invasive, spatially resolved metabolic monitoring of living cell cultures. Our microreactor platform generates controlled oxygen and nutrient cues while simultaneously acquiring label-free Raman spectra, revealing extracellular metabolic fingerprints linked to cell catabolism (e.g., glucose and lactate shifts) and acidification. Analysis across four cell lines uncovered temporal evolution as the dominant source of metabolic variance, while spatial heterogeneity along oxygen gradients is a secondary factor. In particular, diffusion-limited regions exhibited localized acidification and accumulation of stress biomarkers—such as the release of nucleotides—features that cannot be detected using conventional bulk assays. By providing a versatile platform for real-time mapping, this work enables the mechanistic dissection of cell adaptation to microenvironmental stress and supports the prediction of metabolic signatures underlying drug response and treatment outcomes.

## 1. Introduction

In solid tumors, limited and irregular vascularization, together with the high metabolic demands of proliferating tumor cells, lead to the formation of spatial gradients in oxygen and nutrients [1]. Consequently, distinct metabolic zones are formed—ranging from well-perfused, oxygenated regions near blood vessels to more distal hypoxic and nutrient-deprived areas—each applying different selective types of stress on resident cells [2]. To adapt and survive in these conditions, cancer cells undergo metabolic reprogramming, including enhanced glycolysis, lactate accumulation, and active regulation of extracellular pH. These changes contribute not only to survival under stress, but also to immunosuppression [3], invasiveness [4], and resistance to therapy [5].

These metabolic adaptations are spatially and temporally regulated, reflecting the dynamic interplay between tumor cells and their microenvironment. However, such metabolic cues in tumor niches cannot be adequately reproduced in standard 2D cell culture systems, which typically provide uniform conditions throughout the culture and fail to replicate the physiological gradients present in vivo [6]. Although more advanced models (such as microfluidic platforms, co-culture systems and patient-derived tumor models) provide improved physiological relevance and are increasingly used to study cellular interactions and treatment response, they typically rely on labelled assays and endpoint measurements to assess metabolic activity [7,8,9].

To tackle these limitations, Raman spectroscopy has emerged as a powerful alternative, offering molecular profiling based on inelastic light scattering without the need for dyes or reporters [10]. It enables direct detection of key metabolites such as glucose and lactate, or even pH changes reflected in shifts in vibrational signatures [11]. In this context, recent advances have underpinned the versatility of Raman spectroscopy for dynamic metabolic monitoring across diverse extracellular milieus. Notably, Raman-based approaches have been employed to register extracellular metabolic fluctuations, revealing distinct secretory profiles [12,13,14,15]. However, most of these breakthroughs have been achieved either through at-line measurements in large-scale bioreactor (10 L) systems using fiber-optic probes, which sacrifice spatial resolution at cellular scales [16], or through bulk off-line endpoint assays at smaller scales (milli and microreactors) [17]. Current platforms still fall short of providing integrated systems that simultaneously reproduce physiologically relevant gradients and enable real-time, spatially resolved metabolic monitoring [18].

To bridge this gap, we adapted and integrated a microreactor system—building on the developments proposed by Carmona-Fontaine et al.—with Raman spectroscopy to enable continuous, spatiotemporal tracking of extracellular metabolites [19]. Our platform generates oxygen and nutrient gradients from a lateral reservoir while maintaining optical access for Raman measurements, thereby enabling the study of metabolic adaptation under controlled microenvironmental stress. To demonstrate broad applicability, we performed metabolic profiling across diverse cellular models: HEK-293T cells, MDA-MB-231 breast cancer cells, and RCC4 renal cancer cells, including both wild-type and genetically modified variants (RCC4 KO). Raman spectra were acquired from spatially defined regions over four days, and multivariate analysis was applied to extract dominant metabolic patterns. Across all cell models, we detected progressive glucose consumption, lactate secretion, extracellular acidification, and stress-related spectral changes in response to increasing microenvironmental stress. Overall, this platform offers a simple, non-invasive approach for spatiotemporal monitoring of extracellular metabolic profiles in physiologically relevant and evolving microenvironments.

## 2. Materials and Methods

### 2.1. Device Design and Fabrication

The custom 3D-printed culture chamber compatible with Raman spectroscopy was inspired by the MEMIC platform previously published [19] to study oxygen and nutrient gradients in cell cultures. In our version, we designed a lateral reservoir positioned at one end of the chamber. This modification allowed for the controlled diffusion of oxygen and fresh nutrients into the culture medium, while confining the cell layer and its secreted metabolites to an optically accessible chamber. The revised design was essential to ensure that Raman measurements were restricted to the extracellular space directly surrounding the cells, minimizing interference from overlying bulk medium. The custom device was designed using computer-aided design (CAD) software (version 2025 SP5.0) (SolidWorks, Dassault Systèmes, Vélizy-Villacoublay, France) and fabricated by fused filament fabrication (FFF) using polylactic acid (PLA) filament (Ultimaker B.V., Utrecht, The Netherlands). The printed structure measured 15 mm in length, 1.2 mm in height and 6 mm in width (Figure S1, Supporting Information), and featured a reservoir open to air and fresh medium. This configuration enabled the formation of physiologically relevant gradients along the chamber’s longitudinal axis. After printing, the device was sealed between a glass coverslip on top (No. 1, dimensions: 18 × 18 mm, VWR) and a coverslip on the bottom (No. 1.5H, 22 × 22 mm, VWR) using UV-curable adhesive (NOA68, Norland Products Inc., Cranbury, NJ, USA) and cured under a long-wave UV lamp (Thermo Fisher Scientific, Waltham, MA, USA) for 30 min. To maintain sterility and permit gas exchange, a 3D-printed PLA lid was placed over the reservoir during cell culture and Raman measurements.

### 2.2. Cell Culture

HEK-293T cells (CRL-3216™) and MDA-MB-231 (HTB-26TM) cells were purchased from the American Type Culture Collection (ATCC, Manassas, VA, USA). RCC4 cells (cat#03112702) were obtained from the European Collection of Authenticated Cell Cultures (ECACC, Salisbury, UK). The isogenic HIF2α knockout (KO) RCC4 cell line (i.e., RCC4 KO) was generated in-house using CRISPR-Cas9. These cell lines were selected to represent distinct metabolic states relevant to validating the device: HEK 293T as a well-established human epithelial line suitable as a reference model due to its stable phenotype and reproducible metabolism; MDA-MB-231 as a highly glycolytic cancer model; and RCC4 together with its HIF2α-KO variant to assess the influence of HIF2α signaling on extracellular metabolite dynamics. All cell lines were grown under sterile conditions in complete medium, containing high glucose (4.5 g/L) Dulbecco’s Modified Eagle Medium (DMEM, GibcoThermo Fisher Scientific, Waltham, MA, USA) and supplemented with 10% FBS (Gibco, Thermo Fisher Scientific, Waltham, MA, USA, 10270-106) and 1% penicillin-streptomycin (Gibco, Thermo Fisher Scientific, Waltham, MA, USA, 15140-122) unless otherwise specified. Cells were incubated in a 37 °C, 5% CO_2_ incubator and were routinely tested for mycoplasma contamination. For proof of concept experiments, HEK-293T and MDA-MB-231 cells were seeded in 6-well plates in complete DMEM medium and incubated either in normoxia (21% O_2_) or hypoxia (1% O_2_, 5% CO_2_), balanced with N_2_ until 80–90% confluence.

### 2.3. Cell Proliferation Assay

Cell proliferation was evaluated using the MTT (3-(4,5-dimethylthiazol-2-yl)-2,5-diphenyltetrazolium bromide) assay. Cells were seeded in 96-well plates at a density of 2 × 10^3^ cells per well and allowed to attach for 24 h. Subsequently, plates were incubated under normoxic (21% O_2_) or hypoxic (1% O_2_) conditions for up to four days. At each time point (days 1–4), MTT reagent was added to the wells and incubated for 3 h at 37 °C. Formazan crystals were dissolved in DMSO, and absorbance was measured at 570 nm using a Halo LED 96 plate reader (Dynamica Ltd., Livingston, UK; *n* = 3).

### 2.4. Cell Seeding in the Device

Prior to use, devices were UV-sterilized and coated with poly D lysine (Sigma-Aldrich, St. Louis, MO, USA; P6407) to enhance cell adhesion. Cells were seeded at 1 × 10^6^ cells/mL in phenol red-free DMEM (Gibco, Thermo Fisher Scientific, Waltham, MA, USA, 21063029) and incubated for up to 96 h in a 37 °C, 5% CO_2_ incubator. Reservoirs were filled with 500 μL of fresh complete medium, and the device was maintained in a humidified Petri dish to minimize evaporation. To prevent disruption of the culture chamber, liquids were added and removed by orienting the device appropriately and pipetting carefully to avoid air bubbles. Control devices without cells were prepared and maintained under identical conditions, including the same culture medium, incubation period, and Raman measurement schedule, to account for potential spectral changes in the medium over time in the absence of cellular activity.

### 2.5. Fluorescence Microscopy

HEK-293T cells were seeded as described previously and cultured for two days at 37 °C in a humidified incubator with 5% CO_2_. The culture medium was replaced with fresh growth medium containing the Image-IT Green Hypoxia Reagent (Thermo Fisher Scientific, Waltham, MA, USA, 14834) at a final concentration of 5 µM, and cells were incubated for 3 h at 37 °C to allow dye accumulation under low-oxygen conditions. The nuclei were stained with Hoechst 33342 (Thermo Fisher Scientific, Waltham, MA, USA, 62249) for 10 min at room temperature protected from light, followed by incubation with propidium iodide (PI) solution (Sigma Aldrich, St. Louis, MO, USA; 04511) for 15 min at 37 °C to assess cell death. Prior to image acquisition, cells were washed three times with PBS and fixed with 4% paraformaldehyde for 15 min at room temperature. Images were acquired using a Zeiss Axio Observer epifluorescence microscope (Zeiss, Cambridge, UK) equipped with DAPI, GFP and Texas Red filter sets corresponding to Hoechst, Image-IT Green Hypoxia Reagent and PI staining, respectively. Images were captured under identical exposure settings and analyzed using Zeiss Zen blue 3.13 software. Based on the resulting spatial gradients in hypoxia and cell viability along the longitudinal axis of the chamber, three regions were defined for subsequent Raman analysis: z1, adjacent to the reservoir, characterized by low hypoxia signal and high cell viability; z2, an intermediate zone with increased hypoxia signal but maintained viability; and z3, the distal region exhibiting strong hypoxia signal and increased cell death.

### 2.6. Raman Measurements

Raman measurements were performed with an inVia Qontor confocal Raman microscope (Renishaw plc, Wotton-under-Edge, UK). Laser wavelength and output power were set to 785 nm and 73 mW, respectively. Raman spectra were collected daily over four days from zones z1, z2 and z3 using a 10× objective and an 1800 lines/mm grating centered at 850 cm^−1^, covering the spectral region of 600–1100 cm^−1^. Fifteen spectra per zone were acquired with 20 accumulations of 1 s each, focusing 0.4 mm above the cell layer to capture extracellular metabolite signals. For the proof-of-concept analysis, spectra were acquired from supernatants using a 10× objective and a 1200 lines/mm grating centered at 1200 cm^−1^. Ten spectra per condition were recorded using 20 accumulations of 1 s each.

### 2.7. Data Processing

Spectra were preprocessed using a custom Python pipeline (Python v3.X, https://www.python.org, accessed on 27 April 2026). Preprocessing steps included cosmic ray removal, low-pass filtering, background correction, scaling and averaging. After cosmic ray removal, following Wire software 4.4, spectra were first smoothed using a Savitzky–Golay filter (window length 35, polynomial order 2) to reduce high-frequency noise while preserving peak shape [20]. These parameters were selected empirically to balance noise suppression and preservation of metabolic peak profiles, as narrower windows retained excessive noise while wider windows distorted spectral features. Baseline correction was performed using an asymmetric least squares (ALS) algorithm (λ = 10^−6^, *p* = 0.01); these values were iteratively adjusted until sequential changes in key metabolic peaks were consistently resolved across all time points and cell lines without introducing artificial features. After smoothing, baseline correction was performed using an asymmetric least squares (ALS) algorithm implemented with sparse matrices (10 iterations, λ = 10^−6^, *p* = 0.01) [17]. The estimated background was subtracted from each spectrum, and the resulting background-corrected spectra were centered by their mean intensity to minimize global intensity fluctuations. All steps were applied independently to each spectral column. Principal component analysis (PCA), ANOVA–simultaneous component analysis (ASCA), and partial least squares (PLS) were applied to identify multivariate patterns associated with time progression, nutrient availability, and the spatial position of each measurement within the device.

## 3. Results and Discussion

To investigate how cells adapt to defined oxygen and nutrient gradients over time, we developed a 3D-printed microculture platform and adapted it for compatibility with Raman spectroscopy. By employing inexpensive materials in the microreactor fabrication, we sought to achieve an affordable system suitable for high-throughput measurements across multiple cell lines (HEK-293T, MDA-MB-231, RCC4, and RCC4 KO) under diffusion-limited conditions over four days. The following results demonstrate the platform’s capability for spatially and temporally resolved monitoring of extracellular metabolic profiles in live-cell cultures.

### 3.1. Design and Validation of Raman-Compatible Gradient Culture Microreactor

**Figure 1:**
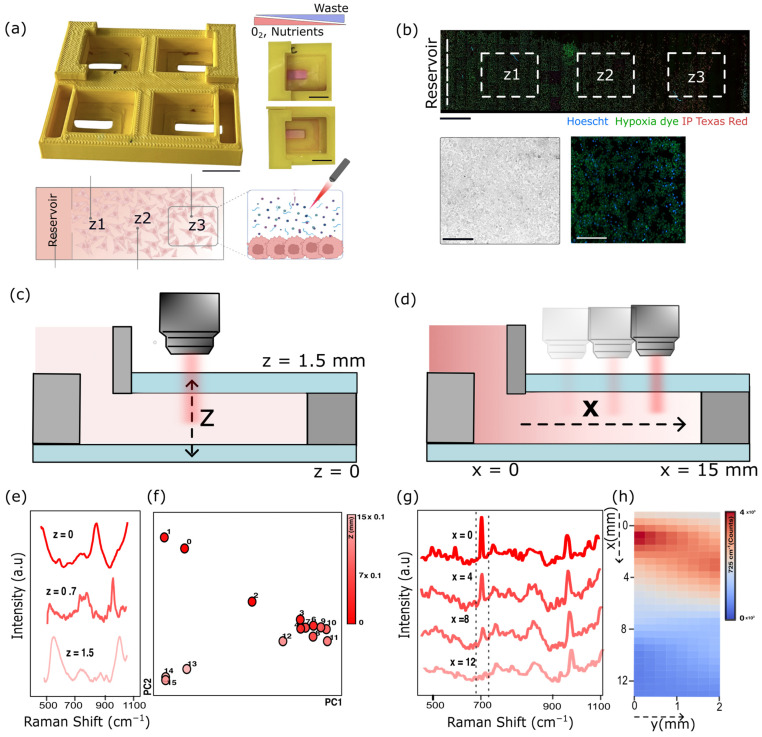
(**a**) Schematic of the 3D-printed microreactor showing the central culture chamber and the lateral reservoir that establishes oxygen and nutrient gradients through passive diffusion. Insets: photographs of the printed device filled with phenol red-containing medium, with or without cells. Below, schematic of the microfluidic device and Raman measurement workflow. Scale bars = 8 mm. (**b**) Fluorescence imaging of HEK-293T cells after two days of culture reveals spatial heterogeneity in viability (IP Texas Red) and hypoxia (Hypoxia Green), defining three regions along the *x*-axis: z1 (normoxic, nutrient-rich), z2 (hypoxic) and z3 (necrotic). Scale bar: 1 mm. Brightfield and fluorescence images of z_2_ cells under hypoxia, showing widespread hypoxia marker signal (green) and nuclear staining with Hoechst (blue). Scale bar: 100 µm. (**c**) Schematic of the *z*-axis scan across the chamber depth, indicating the focal plane used for Raman acquisition. (**d**) Schematic of *x*-axis measurements along the longitudinal direction of the chamber used to assess diffusion from the reservoir (**e**) Representative Raman spectra collected at different focal depths (*z* = 0–1.5 mm). (**f**) PCA score plot of the *z*-scan dataset showing clustering of spectra acquired within the central region and signals originating from the glass windows. (**g**) Raman spectra acquired along the *x*-axis after 10 min of adenine diffusion from the reservoir, showing the characteristic 725 cm^−1^ peak decreasing with distance. (**h**) Heatmap representation of adenine intensity confirming the formation of a diffusion-driven concentration gradient within the chamber.

Building upon previous gradient-generating culture systems, we developed a custom 3D-printed microreactor compatible with Raman spectroscopy (Figure 1a). Each unit fabricated using PLA filament consists of a central culture chamber (200 µL) and a lateral reservoir (600 µL) designed to establish oxygen and nutrient gradients through passive diffusion (Figure S1, Supporting Information). The central chamber is sealed between two glass coverslips to serve as optical windows that enable Raman measurements of extracellular metabolites within the confined culture space (see Section 2 for microreactor printing and sealing). This configuration recreates the microenvironmental heterogeneity characteristic of solid tumors, where steep gradients of oxygen, nutrients, and waste arise due to limited diffusion and intense metabolic activity [21,22]. Experiments shown in Figure 1a (left) illustrate the chamber filled with cell culture medium containing phenol red as a visual indicator, both without cells (top) and with cells (bottom). Notably, a yellowish gradient develops over time as a result of cellular metabolic activity within the chamber. A distinctive improvement over previous designs is the relocation of the reservoir outside the optical path, which allows direct measurement of metabolic fluctuations within the inner chamber without interference from the fresh medium that typically overlays the sample in conventional setups. To maintain sterility throughout the experiment, a custom lid cover was incorporated into the microreactor prototype (as depicted in Figure S1) [19,23,24].

The compatibility of the microreactor with cell culture (HEK-293T) was first validated using optical and fluorescence imaging (Figure 1b) following cell seeding (see Section 2), confirming proper attachment of cells to the bottom glass surface of the central chamber (Figure 1b, insets). It is noteworthy that after two days of culture within the chamber, distinct spatial patterns emerged that correspond to the gradients of cell viability (IP Texas Red) and hypoxia (Hoechst and Hypoxia Green dye). Based on these observations, three zones were defined along the *x*-axis for Raman spatial analysis: zone1, *z*1, adjacent to the reservoir (normoxic and nutrient-rich); *z*2, the intermediate zone (hypoxic); and *z*3, the distal zone (necrotic and nutrient-depleted).

It is worth mentioning that we opted for standard glass slides as Raman windows—an affordable alternative to quartz or CaF_2_, which are commonly used in such setups but incompatible with high-throughput device fabrication—due to the high price for single-use consumables. Although glass allows efficient light transmission, it exhibits intrinsic Raman and fluorescence background signals, particularly above 1300 cm^−1^. To address this, we (1) focused our Raman fingerprint analysis on the spectral range of 500–1200 cm^−1^, which covers the main vibrational modes of key metabolites while avoiding glass-related bands, and (2) adjusted the focal plane to the axial center of the sample volume to minimize interference from the glass surfaces and remain as close as possible to the cell layer (Figure 1c). Under these focusing conditions, the Rayleigh range of the Gaussian beam, defining the axial extent over which the beam remains tightly focused, is confined to the extracellular medium, so that Raman scattering from the glass substrate and coverslip is effectively excluded from the collection volume. To carry out this study, we assembled the device using two glass slides from different manufacturers, each with slightly distinct Raman signatures (Figure 1e). This design allowed us to clearly differentiate whether spectral interferences originated from the top or bottom glass surfaces, or from a combination of both. A vertical *z*-scan was performed from the bottom (0 mm) to the top glass slide (1.5 mm) with a step size of 100 µm, yielding a dataset of 16 individual spectra across the *z*-axis of the inner chamber filled with cell culture medium. When analyzed by principal component analysis (PCA) in Figure 1f, the spectra collected near the slides showed characteristic peaks corresponding to the window materials (opposite values in PC2 depending on slide manufacturer), whereas those acquired within the central region of 300–1200 µm clustered together in the plot, indicating minimal glass interference and revealing the characteristic spectral features of the culture medium (Figure 1e, *z* ≈ 0.7 mm). Based on these findings, a focal height of *z* = 0.4 mm was selected for all subsequent Raman measurements (see Figure S2 for spectra overlay). On top of this, a key feature of the device is its ability to generate gradients by simple diffusion from the reservoir into the inner chamber, where cells actively consume nutrients. To validate this mechanism in a straightforward configuration, adenine (1 mM) was added to the reservoir, and Raman spectra were acquired along the *x*-axis after 10 min (Figure 1d). The characteristic Raman peak of adenine at 725 cm^−1^ was clearly detectable near the reservoir and progressively decreased in intensity with increasing distance, which is consistent with diffusion-limited transport (Figure 1g) [25]. This spatial distribution was further visualized as a heatmap, confirming the generation of a concentration gradient across the chamber (Figure 1h), in agreement with the diffusion simulation presented in Supplementary Figure S3.

### 3.2. Temporal and Spatial Metabolic Profiling in the 3D-Printed Gradient Device

**Figure 2:**
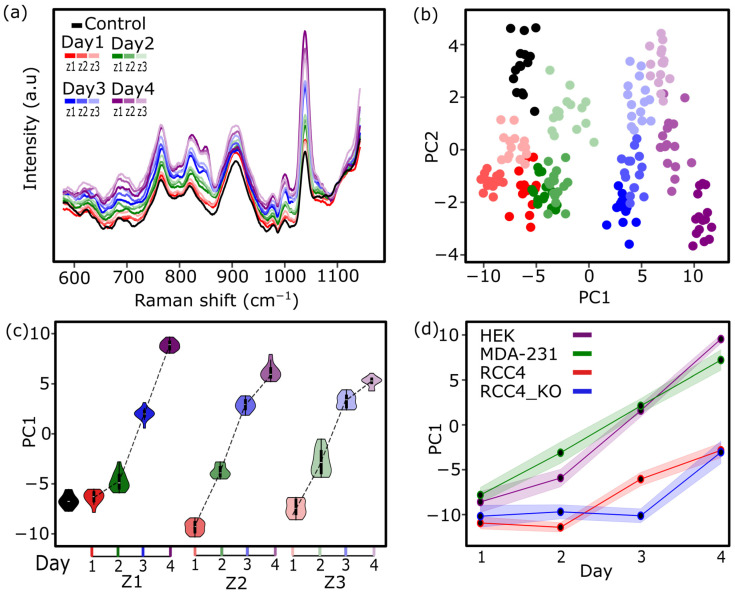
(**a**) Average Raman spectra (600–1200 cm^−1^) of HEK-293T cells over four days across three spatial zones: z1 (well-perfused), z2 (hypoxic), and z3 (necrotic). (**b**) PCA plot showing temporal evolution along PC1 and spatial separation along PC2; black dots indicate control medium without cells. (**c**) PC1 scores by zone and day reveal distinct temporal trends: stable in z_1_, linear increase in z_2_, and a progressive flattening in z_3_ consistent with nutrient depletion and reduced viability. (**d**) Comparative PC1 trajectories for four cell lines—HEK-293T, MDA MB 231, RCC4, and RCC4-KO—highlight differences in metabolic dynamics, with stronger PC1 increases in HEK-293T and MDA-MB-231. *n* ≥ 3 biological replicates for all experiments.

To investigate metabolic fluctuations under diffusion-limited conditions in our microreactor, HEK-293T cells were initially cultured for four consecutive days, and Raman spectra were acquired across the three spatial zones *z*1, *z*2, and *z*3 as defined above. Average spectra between 600 and 1200 cm^−1^ in Figure 2a revealed subtle vibrational shifts over time and across regions, reflecting spatiotemporal changes in the extracellular milieu. PCA was applied to the spectral dataset (Figure 2b) to reduce dimensionality and identify dominant patterns in spectral variations, which are projected onto the first two principal components (black dots represent control conditions without cells). The first principal component (PC1), accounting for most of the variance, primarily captured temporal progression, with a clear shift in spectral profiles from day 1 to day 4. In contrast, the second component (PC2) reflected for the most part the spatial separation between zones, particularly at later time points. With the aim to better represent such variability among spectra, PC1 scores were plotted in Figure 2c by spatial zone and day, and distinct region-specific temporal patterns were observed. In *z*1, PC1 values remained relatively stable between days 1 and 2, followed by a gradual increase at later time points. In *z*2, PC1 increased linearly over time, whereas in *z*3, values reached a semi-plateau at days 3 and 4, suggesting metabolic stress and reduced cell viability. These findings indicate that PC1 trajectories may reflect underlying metabolic activity and proliferation dynamics of the cultured cells. Hence, the same Raman analysis was repeated for all four cell lines: in addition to HEK-293T and MDA-MB-231 cells, two RCC4-derived lines were included: RCC4 wild-type (RCC4) and RCC4 HIF2α knockout (RCC4 KO) generated via CRISPR–Cas9 (see Figures S4–S6 for the spectra dataset for each cell line). As shown in Figure 2d, MDA-MB-231 and HEK-293T cells exhibited a marked increase in PC1 (collected in *z*2) over time, consistent with higher metabolic activity. In contrast, RCC4 and RCC4 KO cells displayed slower PC1 shifts, likely reflecting more modest metabolic changes. This trend was corroborated by MTT assays: under normoxic conditions (Figure S7a, Supporting Information), HEK-293T and MDA-MB-231 cells showed the highest proliferation rates. Collectively, these results support the assumption that PC1 captures metabolic differences associated with cell growth behavior, which vary among cell types and oxygen conditions.

### 3.3. Temporal Metabolic Adaptation in HEK-293T Cells

**Figure 3:**
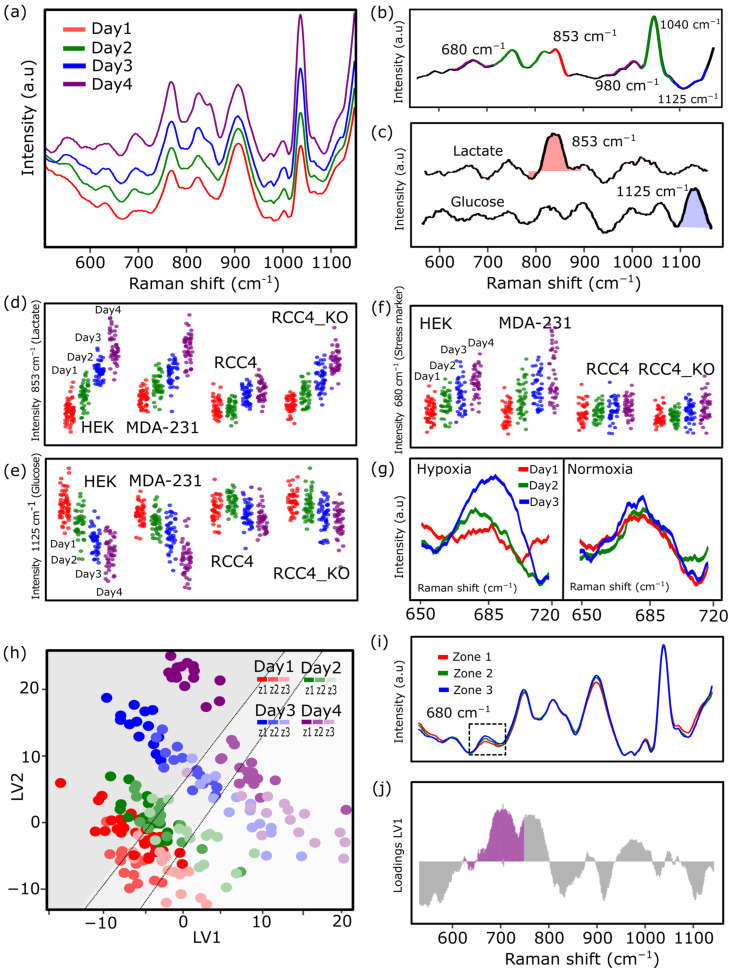
(**a**) Average Raman spectra of HEK-293T cells over four days. (**b**) ASCA loading plot with main peaks contributing to temporal variation. (**c**) Reference spectra of glucose and lactate. Temporal evolution of lactate (**d**) and glucose (**e**) peak intensities across four cell lines. (**f**) Temporal evolution of the 680 cm^−1^ stress-related vibration. (**g**) Validation of the 680 cm^−1^ band under controlled oxygen conditions. (**h**) PLS analysis combining temporal and spatial data, with LV1 and LV2 projections showing separation by time and spatial zone. (**i**) Average Raman spectra per zone (z1–z3) highlighting an increased 680 cm^−1^ signal in *z*. (**j**) LV1 loading plot confirming contributions from 680 cm^−1^. *n* ≥ 3 biological replicates for all experiments.

Progressive spectral shifts across days suggested the accumulation or depletion of specific extracellular metabolites. We therefore extended our analysis beyond the PC1 trajectories to identify the key molecular features underlying these temporal metabolic changes in HEK-293T cells (Figure 3a). As shown in Figure 3a, an ANOVA–Simultaneous Component Analysis (ASCA) was performed, integrating data from all three spatial regions to isolate the variance specifically associated with the time factor. The time component was calculated by subtracting the global mean spectrum and determining, for each day, the deviation of its mean spectrum from this overall mean. PCA was then applied to this time submatrix to extract the dominant temporal patterns, yielding ASCA score trajectories and corresponding loading spectra. Loading plots (Figure 3b) revealed that changes in PC1 were primarily driven by peaks at approximately 680, 853, 980, 1040, and 1125 cm^−1^. Reference spectra obtained from aqueous glucose and lactate solutions (Figure 3c) supported the assignment of the 1125 cm^−1^ peak to glucose and the 853 cm^−1^ peak to lactate, which is consistent with previously reported Raman signatures for these metabolites in biological samples [11,26].

To assess the qualitative reliability of these assignments, calibration curves were constructed using serial dilutions of glucose and lactate. The resulting peak intensities exhibited strong linear correlations with concentration (*R*^2^ = 0.95 for glucose and *R*^2^ = 0.98 for lactate), confirming the suitability of these Raman bands for tracking relative metabolite dynamics in cell culture experiments, where the complexity of the biological matrix makes absolute quantification challenging (Figure S8; Supporting Information). Based on these validated assignments, Raman intensity values at 1125 cm^−1^ (glucose) and 853 cm^−1^ (lactate) were extracted from the experimental data and plotted over time in *z*2. While lactate-associated signal intensity increased over time (Figure 3d), the glucose signal decreased (Figure 3e), supporting progressive glycolytic activity and catabolism across all cell lines tested.

In addition to these metabolite-associated peaks, the signal at 680 cm^−1^ was also consistently detected and showed distinct dynamics across cell lines (Figure 3f). HEK-293T and MDA MB 231 cells showed a progressive increase in intensity over time, while RCC4 wild-type and RCC4 KO cells maintained relatively stable levels, with the KO line exhibiting a slight increase at day 4. This variation may reflect differences in cellular or metabolic stress, consistent with previous studies that have attributed this band to stress-associated metabolites such as purine derivatives [27,28] (see Figure S9 for Raman spectra of guanosine).

The increase in this signal under hypoxic conditions may therefore indicate a stress response and lysis accompanying enhanced glycolytic activity, as supported by the concurrent accumulation of lactate. To subsequently validate the presence of this peak under hypoxia, the appearance of the 680 cm^−1^ peak was further examined under controlled normoxic (21% O_2_) and hypoxic (1% O_2_) conditions in T-75 flasks (Figure S10, Supporting Information). A clear increase in intensity was detected under hypoxia at day 3 in HEK-293T cells (Figure 3g), whereas no change was observed under normoxia, supporting the association of this band with microenvironmental stress.

To further resolve spatial and temporal patterns of metabolic adaptation, a Partial Least Squares (PLS)-based zone separation was performed across the entire Raman dataset of HEK-293T cells followed by latent variable analysis. The goal was to determine whether specific vibrational features were more characteristic of particular spatial regions, thereby enabling the classification or probabilistic assignment of spectra to their respective zones. Projection of the data onto the first two latent variables (LV1 and LV2) revealed a clear separation of samples by time, while also capturing spatial variation across zones, as highlighted by the gray-shaded area in Figure 3h. This observation suggests that certain spectral signatures are more characteristic of specific zones, reflecting localized metabolic behaviors within the microenvironment. Furthermore, the Raman spectra from each zone were averaged and compared, repeating the same ASCA approach that had previously been performed with days. Among the features showing differential behavior, the 680 cm^−1^ peak, previously associated with metabolic or oxidative stress, exhibited a notable increase in intensity in *z*3 (Figure 3i). This spatial distribution suggests localized accumulation of stress in diffusion-limited regions of the device. Not only that, the loading plot for LV1 (Figure 3j) also confirmed strong contributions from the 680 cm^−1^ peak, along with other relevant features such as 853 cm^−1^, 980 cm^−1^, 1040 cm^−1^ and 1125 cm^−1^.

### 3.4. Spatial Analysis of Metabolic Variations

Beyond the metabolite-associated and stress-related peaks described above, a strong contribution was also observed in the range of 1040–1050 cm^−1^ across all time points and cell lines in the PC1 loading plot (Figure 3b). Given its constant presence throughout time points and cell lines, this signal was hypothesized to originate from a component of the culture medium rather than from cellular metabolites. Previous studies have reported Raman signals of other biological buffer systems, raising the possibility that this signal may reflect the presence of HEPES buffer in our commercial DMEM media [11,20].

**Figure 4:**
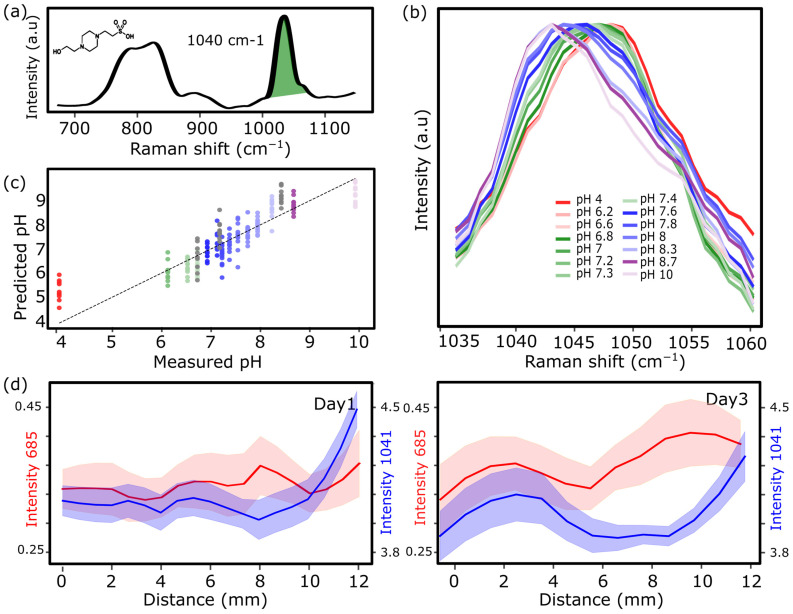
(**a**) Raman spectrum of 1 M HEPES buffer showing a prominent band at 1040 cm^−1^ (green), associated with pH-sensitive vibrational modes of the buffer molecule. (**b**) Raman spectra of HEPES-buffered DMEM adjusted to different pH values (4–10), showing progressive shifts in peak position and intensity of the 1040–1050 cm^−1^ band as a function of pH. (**c**) PLS calibration model correlating measured and predicted pH values using Raman spectra from HEPES-buffered DMEM samples, demonstrating strong agreement (*R*^2^ > 0.95) and robust prediction of independent test data (grey dots). (**d**) Spatial mapping of Raman intensities at 1041 cm^−1^ (pH-associated, blue) and 685 cm^−1^ (stress-associated, red) along the chamber’s *x*-axis (0 = region closest to the reservoir) on day 1 and day 3. The 1041 cm^−1^ signal reveals progressive extracellular acidification over time, while the 685 cm^−1^ peak indicates increasing stress in distal regions.

To investigate this, Raman spectra of pure HEPES buffer (Gibco, 11560496) were acquired, showing a characteristic peak at 1040 cm^−1^ (Figure 4a). To assess whether this HEPES-associated peak was sensitive to pH, Raman measurements were performed on DMEM medium supplemented with HEPES buffer solution adjusted to a pH range of 4 to 10. As shown in Figure 4b, the 1040–1050 cm^−1^ band exhibited progressive shifts in peak position and intensity as a function of pH, consistent with spectral sensitivity to protonation states.

To quantify the relationship between pH and the Raman response at 1040 cm^−1^, a PLS model was constructed using spectra from HEPES-buffered DMEM samples of known pH. As shown in Figure 4c, the calibration curve exhibited a strong linear correlation between the measured pH and the corresponding spectral response. The model also accurately predicted pH values for an independent test dataset (grey dots in Figure 4c) that was not included in the training set, thereby confirming its robustness and predictive reliability.

Finally, the entire chamber was imaged, rather than collecting signals only from predefined regions, following the procedure described in Figure 1g,h, and now performed with cells present. To spatially resolve pH- and hypoxia-associated changes across the device (along the *x*-axis, where 0 corresponds to the region closest to the reservoir), Raman intensities at 1041 cm^−1^ and 685 cm^−1^ were mapped on day 1 and day 3 (Figure 4d). The band at 1041 cm^−1^, previously validated as pH-sensitive, exhibited a modest gradient on day 1 that became more pronounced by day 4, indicating progressive extracellular acidification over time. Similarly, the 685 cm^−1^ signal, associated with hypoxia-induced stress, showed minimal variation on day 1 but increased markedly by day 4 in distal regions. Consistently, plotting PC1 scores as a function of spatial position along the *x*-axis revealed a distinct gradient across the chamber (Figure S11, Supporting Information). These spatial trends highlight the emergence of physiologically relevant microenvironmental gradients under sustained culture conditions in the device and demonstrate the ability of Raman spectroscopy to accurately capture them.

## 4. Conclusions

In summary, this study presents a novel integration of a 3D-printed, gradient-generating cell culture device using Raman spectroscopy to monitor extracellular metabolic dynamics with spatial and temporal resolution. Across models, progressive glucose consumption, lactate secretion, extracellular acidification and stress-related spectral changes were detected in response to increasing microenvironmental stress. These results demonstrate the ability of the microreactor platform to capture physiologically relevant metabolic adaptations under diffusion-limited conditions that resemble the tumor microenvironment.

Beyond confirming known responses to hypoxia and nutrient deprivation, this system provides a versatile analytical framework that, when combined with machine learning methods, enables the study of cellular metabolism in complex microenvironments. We foresee that its compatibility with multiple cell types and label-free readout will make it particularly suitable for longitudinal monitoring, drug testing, and mechanistic investigations of metabolic and stress responses in vitro.

As current limitations, throughput remains constrained by the sequential nature of Raman acquisition, and the closed chamber design does not yet permit spatially resolved supernatant sampling for integration with complementary metabolomics approaches. Addressing these aspects will be the focus of future work.

## 5. Patents

Plou, J.; López, E.; Seifert, A.; Merkens, S.; Márquez, M. Modular multi-well cell culture plate optimized for Raman spectroscopy imaging. PCT Patent Application PCT/EP2025/061297.

## Data Availability

The data that support the findings of this study are available from the corresponding authors upon reasonable request.

## Supplementary Materials

The following supporting information can be downloaded at https://www.mdpi.com/article/10.3390/bios16050266/s1. Figure S1: CAD model of the culture chamber with dimensions in millimeters; Figure S2: Effect of optimal focusing on mitigating glass interference; Figure S3: Comparison between simulated and experimental adenine diffusion profiles; Figure S4: Raman spectra (500–1200 cm^−1^) of MDA-MB-231 cells across all experimental groups (*n* = 3); Figure S5: Raman spectra (500–1200 cm^−1^) of RCC4 cells across all experimental groups (*n* = 3); Figure S6: Raman spectra (500–1200 cm^−1^) of RCC4-KO cells across all experimental groups (*n* = 3); Figure S7: MTT assay showing cell viability in HEK-293T, MDA-MB-231, RCC4, and RCC4 KO cell lines cultured under (a) normoxia (21% O_2_) and (b) hypoxia (1% O_2_) for the indicated time; Figure S8: Calibration curves for (a) glucose and (b) lactate measured in aqueous solution using Raman spectroscopy; Figure S9: Raman spectra of guanosine. Figure S10: Raman spectral profiles of culture supernatants collected from (a) HEK-293T and (b) MDA-MB-231 cells cultured under normoxic (blue) or hypoxic (red) conditions for up to three days; Figure S11: PC1 scores as a function of spatial position after four days of culture.

## Abbreviations

The following abbreviations are used in this manuscript:

ASCA	ANOVA–simultaneous component analysis
DMEM	Dulbecco’s Modified Eagle Medium
FBS	Fetal Bovine Serum
HEPES	4-(2-hydroxyethyl)-1-piperazineethanesulfonic acid
KO	Knockout
PCA	Principal Component Analysis
PLS	Partial Least Squares
