# Supplementary material for: Longitudinal Monitoring of Metabolic Gradients in Microreactor Culture Platforms by Raman Spectroscopy
Source: Biosensors (Basel). 2026 May 2;16(5):266. doi: 10.3390/bios16050266 (PMC13204390; doi:10.3390/bios16050266)
Supplement: Supplementary file 1 [file biosensors-16-00266-s001.zip › biosensors-4249618-supplementary.pdf]

Supporting Information

Article

Longitudinal Monitoring of Metabolic Gradients in Microreactor Culture Platforms by Raman Spectroscopy

Maitane Márquez [1,2,†], Javier Plou [1,†,*], Stefan Merkens [3], Eneko Lopez [1,], Carla Solé [2], Esther Arnaiz [2], Mariana Medina-Sánchez [3,4,5], Charles H. Lawrie [2,5,6,7] and Andreas Seifert [1,5*]

1 Nanoengineering Group, CIC nanoGUNE BRTA, Tolosa Hiribidea 76, San Sebastian 20018, Spain; e-mail@e-mail.com
2 Molecular Oncology Group, Biogipuzkoa Health Research Institute, Paseo Dr. Begiristain s/n, Donostia-San Sebastián 20014, Spain.
3 Nanobiosystems Group, CIC nanoGUNE BRTA, Tolosa Hiribidea 76, San Sebastian 20018, Spain.
4 Center for Molecular Bioengineering (B CUBE), Dresden University of Technology, Dresden 01307, Germany.
5 IKERBASQUE, Basque Foundation for Science, Euskadi Plaza 5, Bilbao 48009, Spain.
6 Radcliffe Department of Medicine, University of Oxford, OX3 9DU Oxford, United Kingdom.
7 Sino-Swiss Institute of Advanced Technology (SSIAT), Shanghai University, Shanghai, 201899, People's Republic of China.
† These authors contributed equally to this work.
* Correspondence: j.plou@nanogune.eu (J.P.); a.seifert@nanogune.eu (A.S.)

Keywords: metabolic heterogeneity; Raman spectroscopy; microreactors; real-time monitoring

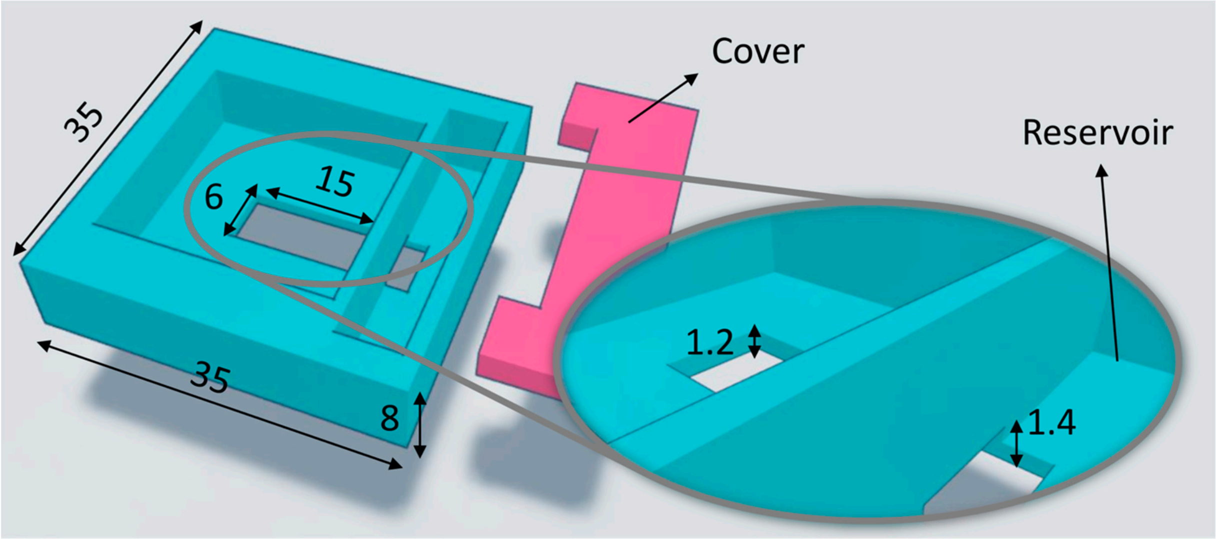

Figure S1. CAD model of the culture chamber with dimensions in millimeters.

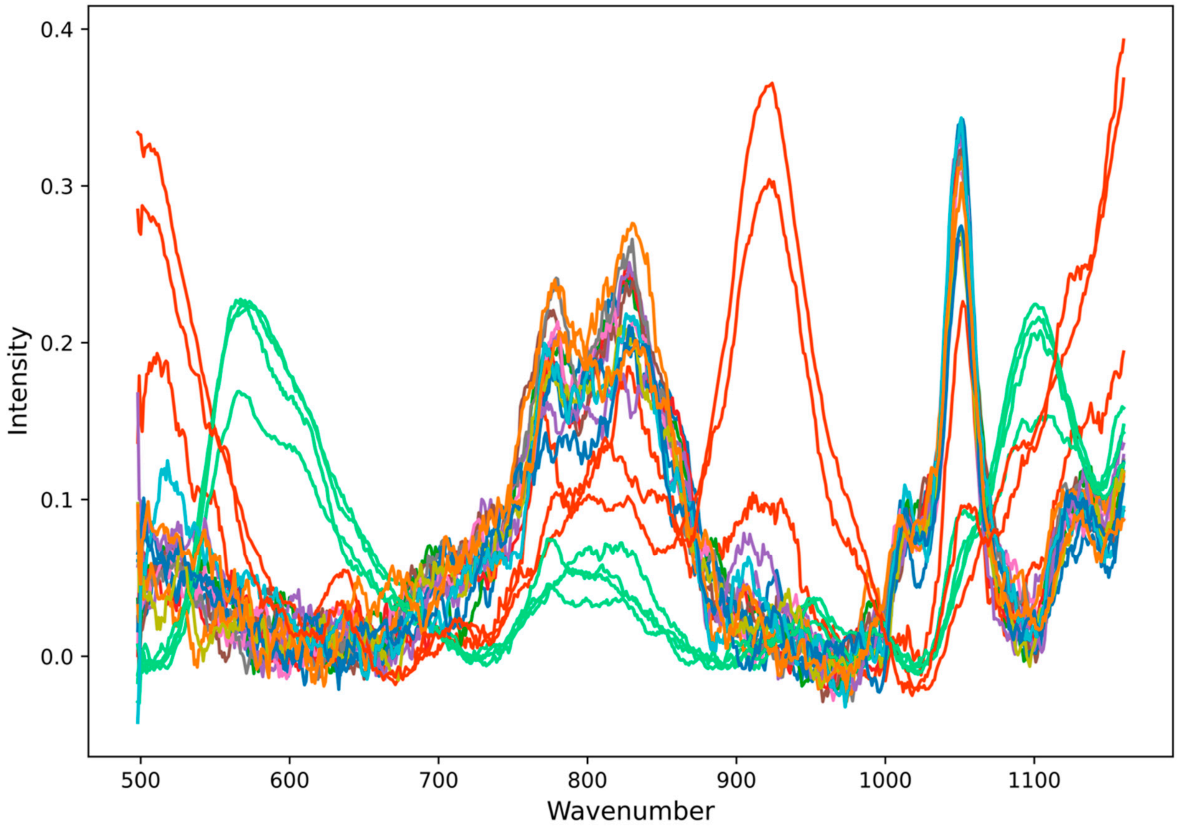

Figure S2. Effect of optimal focusing on mitigating glass interference. Overlay of Raman spectra acquired across the axial depth of the microreactor. The broad, high-intensity curves in red and green represent measurements taken near the bottom and top glass coverslips, respectively, highlighting the strong Raman background of the substrates. In contrast, the tightly clustered spectra in multiple colors correspond to the central region of the chamber (optimal focus). This explicit comparison visually confirms that our spatial focusing strategy effectively isolates the biological and medium-related Raman features from the background materials.

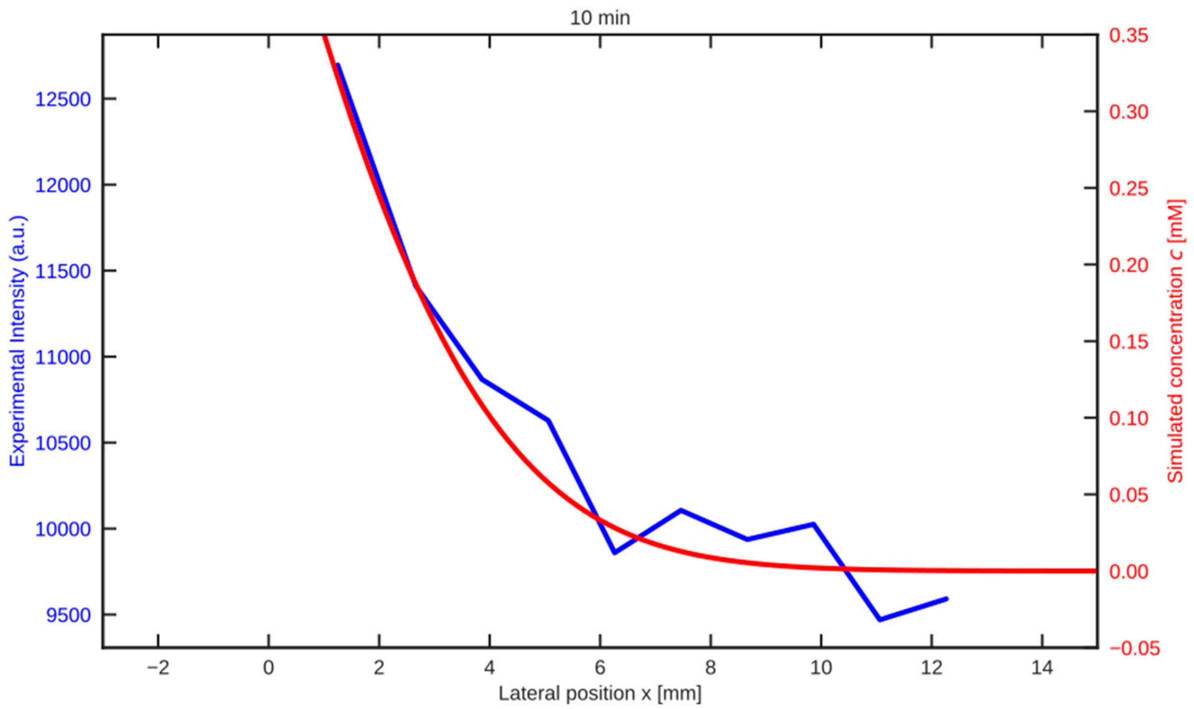

Figure S3. Comparison between simulated and experimental adenine diffusion profiles. A 3D diffusion model of the microreactor was implemented in COMSOL Multiphysics based on the original CAD geometry to simulate lateral diffusion of adenine from the reservoir into the central chamber. The diffusion equation (Fick's second law, $\frac{\partial c_A}{\partial t} = D_A \nabla^2 c_A$) was solved with and initial concentrations of 1 mM (reservoir) and 0 mM (central chamber). No-flux boundary conditions were applied to all surfaces, and mesh convergence resulted in a maximum element size of 0.26 mm. The simulated concentration profile after 10 min (red curve) closely matches the experimentally measured Raman intensity decay at 725 cm^{-1} along the x-axis (blue curve), confirming that the observed signal distribution is governed by diffusion-limited transport.

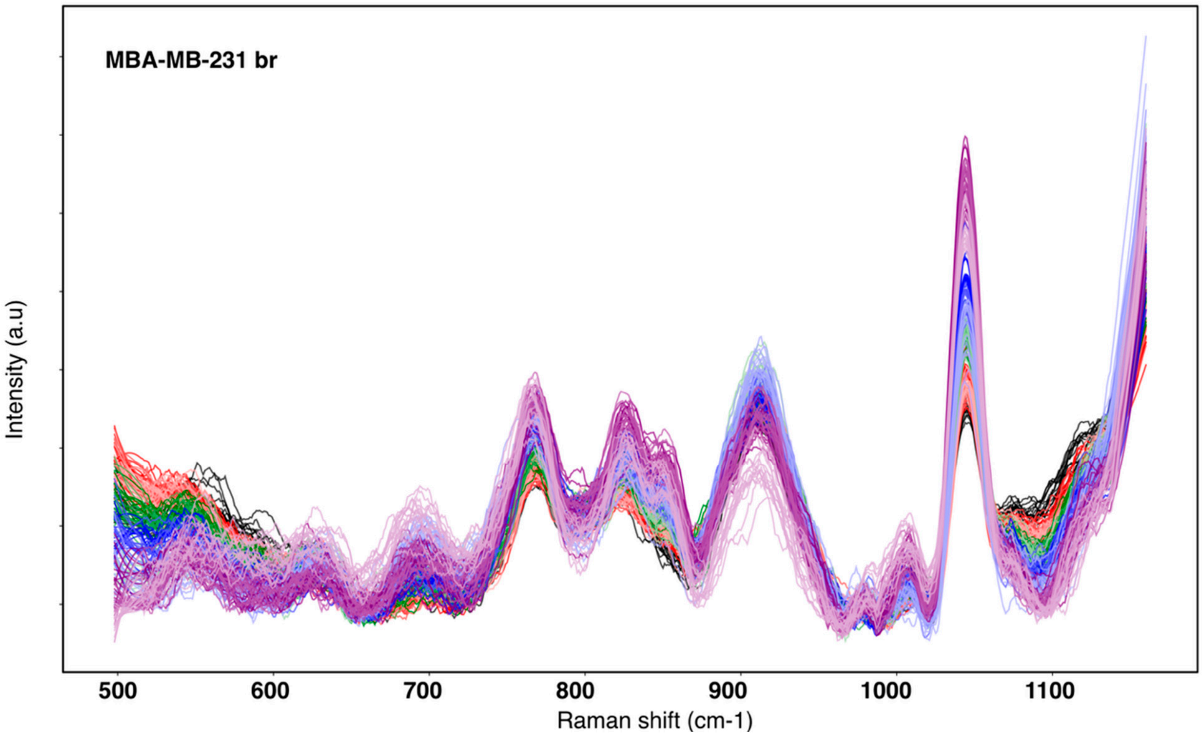

Figure S4. Raman spectra (500 – 1200 cm^{-1}) of MBA-MB-231 cells across all experimental groups (n = 3).

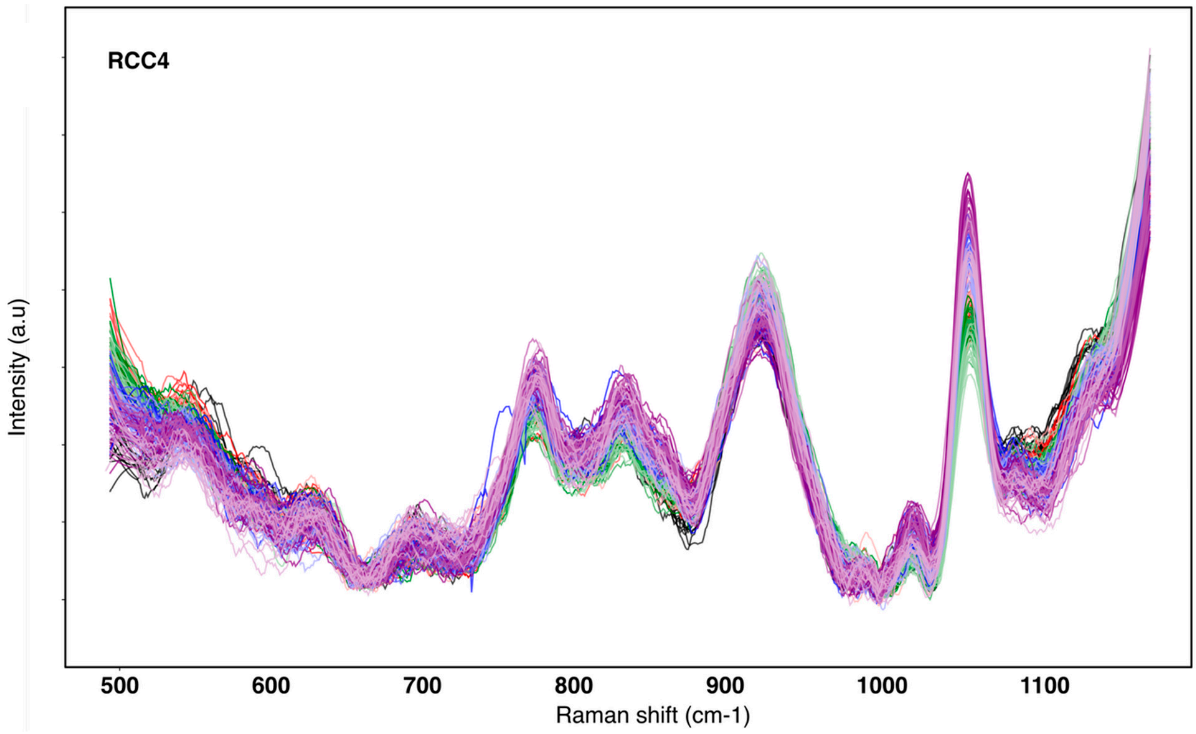

Figure S5. Raman spectra (500 – 1200 cm⁻¹) of RCC4 cells across all experimental groups (n = 3).

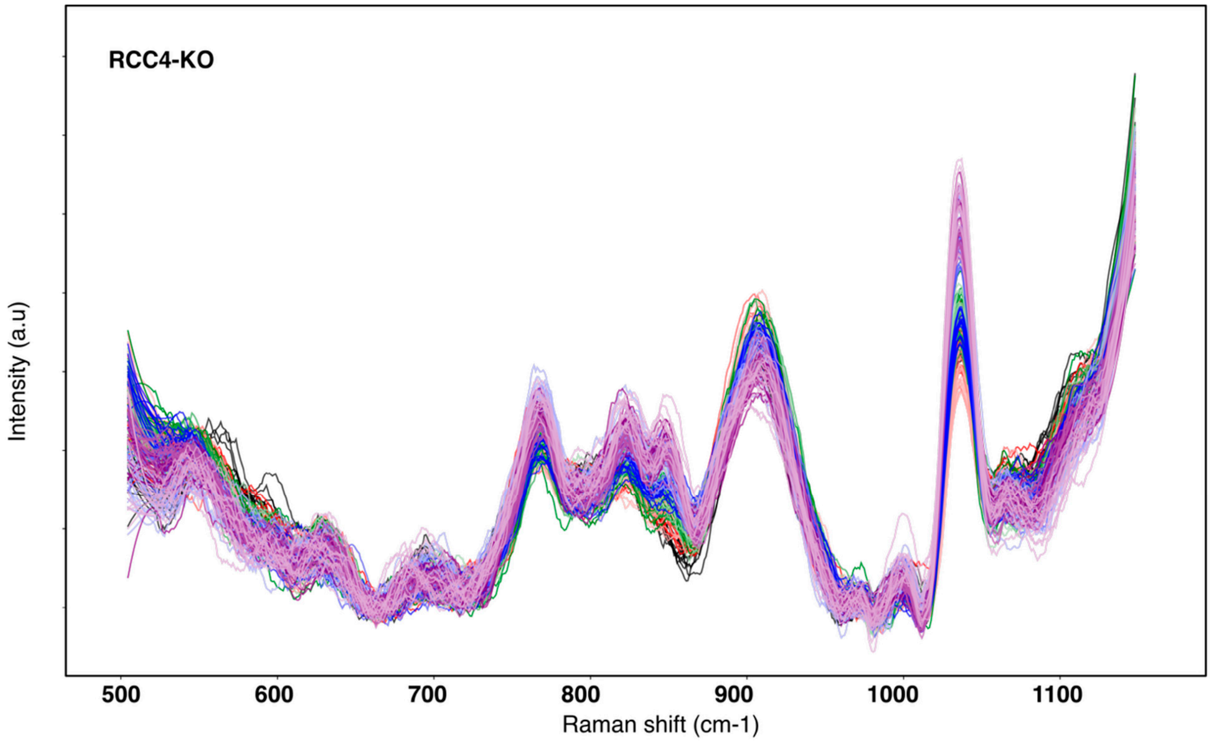

Figure S6. Raman spectra (500 – 1200 cm^{-1}) of RCC4 KO cells across all experimental groups ($n = 3$).

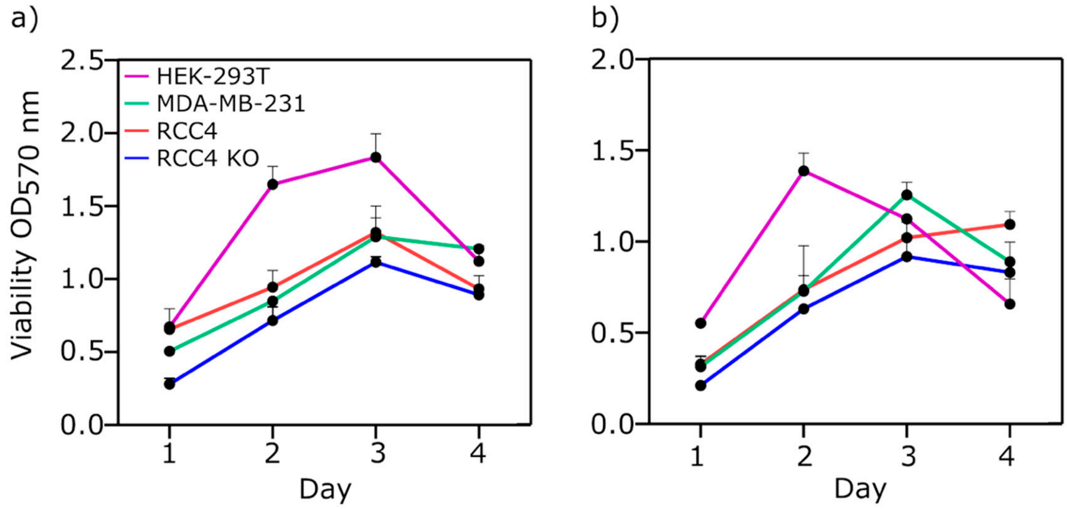

Figure S7. MTT assay showing cell viability in HEK-293T, MDA-MB-231, RCC4, and RCC4 KO cell lines cultured under (a) normoxia (21% O_2) and (b) hypoxia (1% O_2) for the indicated time. Cell viability was determined by measuring absorbance at 570 nm (OD_{570}) and normalizing the values to wells containing medium alone. Data are presented as mean ± SD from 3 independent experiments.

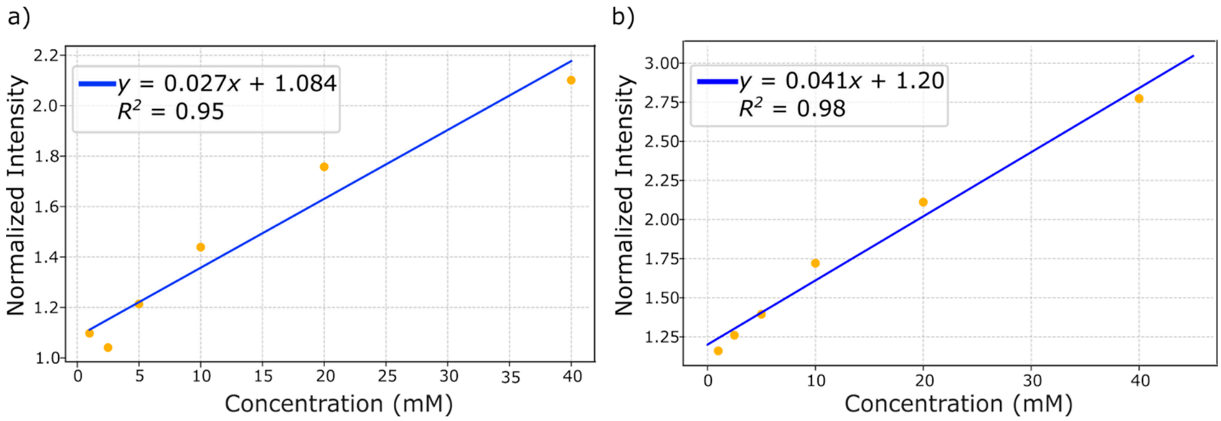

Figure S8. Calibration curves for (a) glucose and (b) lactate measured in aqueous solution using Raman spectroscopy. Standard solutions were prepared at increasing concentrations (0 – 40 mM), and peak intensities at 1125 cm⁻¹ (glucose) and 853 cm⁻¹ (lactate) were plotted against known concentrations.

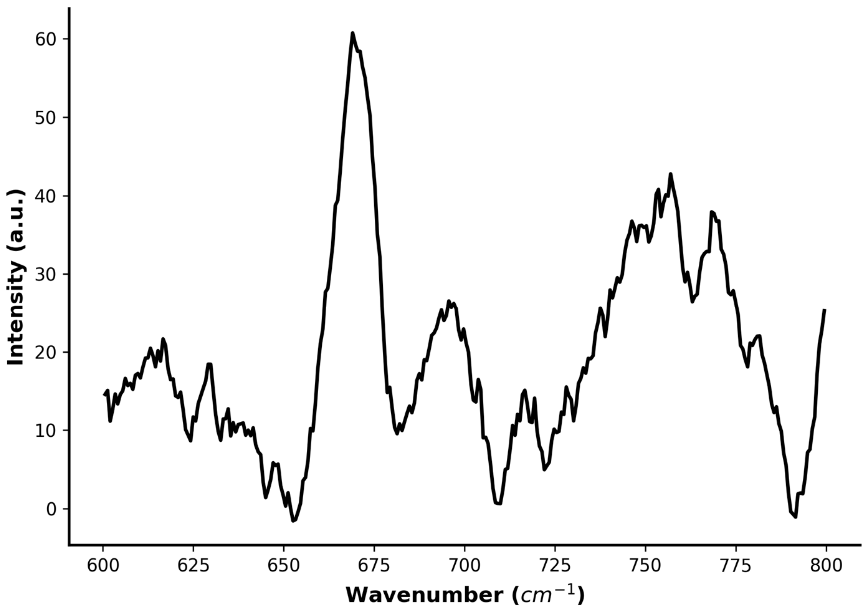

Figure S9. Raman spectrum of 5 mM guanosine in aqueous solution. The principal vibrational band at 670–680 cm^{-1} is characteristic of guanine ring breathing mode.

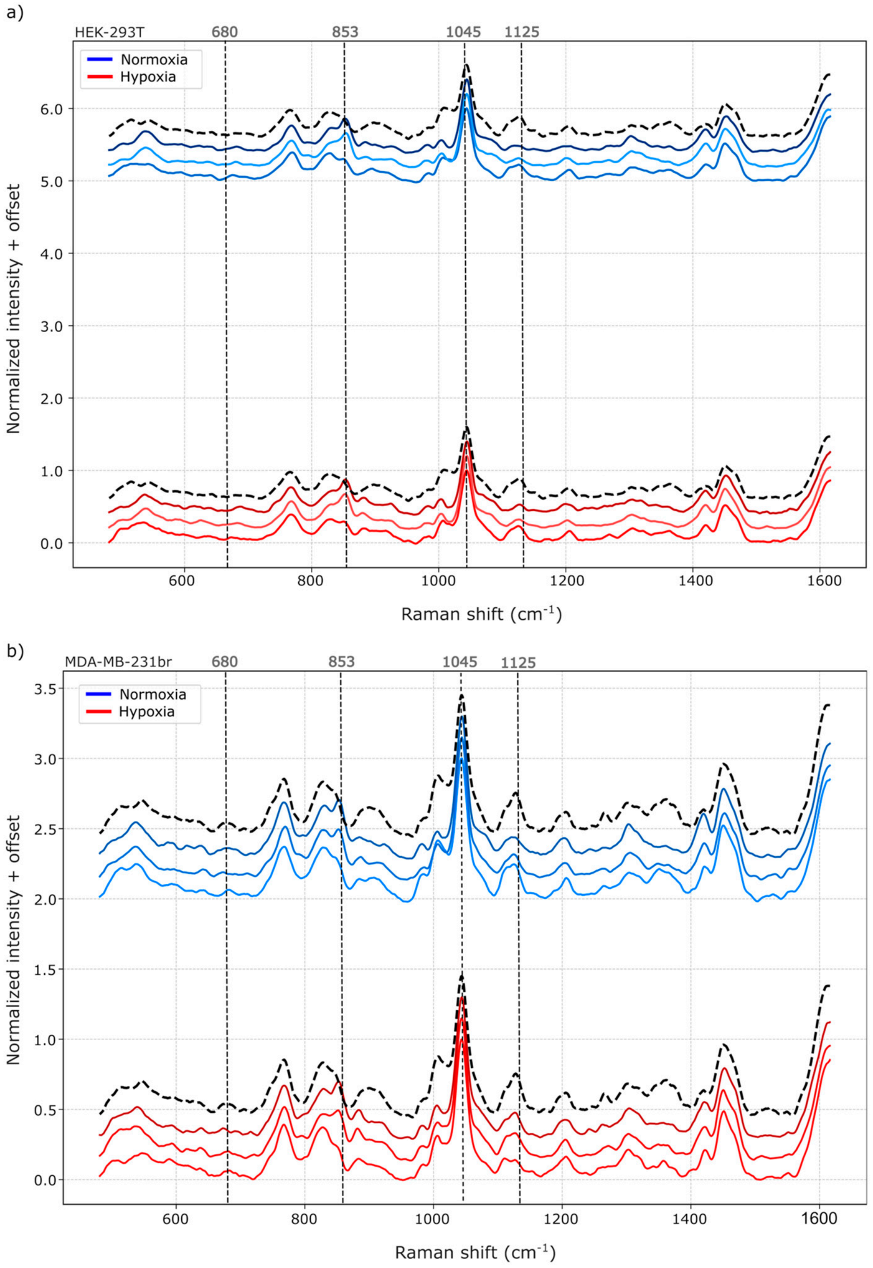

Figure S10. Raman spectral profiles of culture supernatants collected from (a) HEK-293T and (b) MDA-MB-231 cells cultured under normoxic (blue) or hypoxic (red) conditions for up to three days. Vertical dashed lines highlight key Raman shifts associated with cellular or metabolic stress (680 cm^{-1}), lactate (853 cm^{-1}), pH-sensitive region (1045 cm^{-1}), and glucose (1125 cm^{-1}).

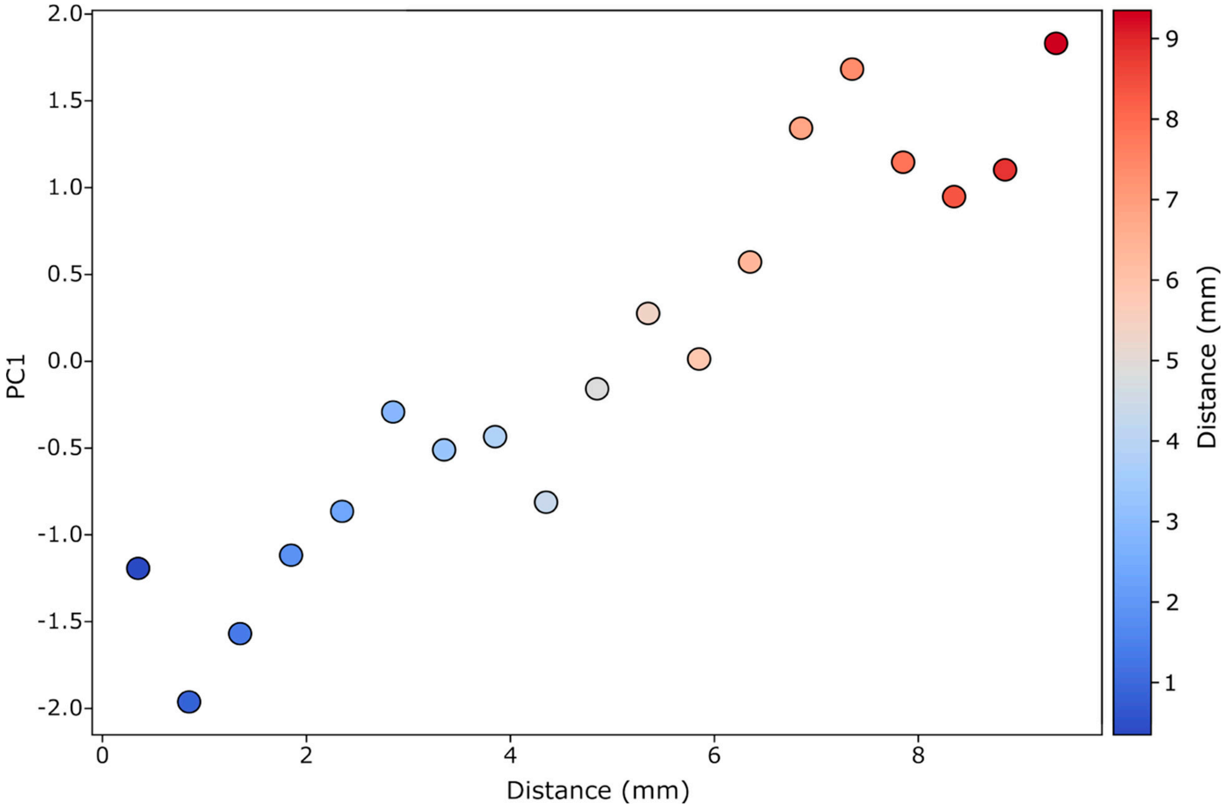

Figure S11. PC1 scores as a function of spatial position after four days of culture.
